# Preparation and Characterization of Intrinsic Low-κ Polyimide Films

**DOI:** 10.3390/polym13234174

**Published:** 2021-11-29

**Authors:** Yu Sun, Tao Li, Haiyang Dai, Manman Wang, Renzhong Xue, Jing Chen, Dewei Liu

**Affiliations:** Henan Key Laboratory of Magnetoelectronic Information Functional Materials, College of Physics and Electronic Engineering, Zhengzhou University of Light Industry, Zhengzhou 450002, China; sy3115@126.com (Y.S.); daihaiyang720@163.com (H.D.); wmanman1@163.com (M.W.); xrzbotao@163.com (R.X.); wulicj@126.com (J.C.); liudw2002@163.com (D.L.)

**Keywords:** polyimide, low dielectric constants, positron annihilation, free volume fraction

## Abstract

Three fluorinated polyimide (PI-FP, PI-FO and PI-FH) films with low dielectric constants and excellent comprehensive properties were successfully prepared using a polycondensation reaction method by incorporating p-phenylenediamine (PDA), 4-4′-diaminodiphenyl ether (ODA) and 4,4′-(Hexafluoroisopropylidene) bis (p-phenyleneoxy) dianiline (HFPBDA) into 4,4′-(Hexafluoroisopropylidene) diphthalic anhydride (6FDA), respectively. The effects of the diamine monomer structure on optical, dielectric and mechanical properties were investigated. Compared with PDA and ODA, HFPBDA can effectively improve the optical and dielectric properties of PI due to due to its special chain structure. Among the three PI films, PI-FH film presents the best optic transmission (highest transmittance = 90.2%) and highest energy gap (2.69 eV). The dielectric properties of PI-FH film improve the most. The dielectric constant and loss at 10^4^ Hz are reduced to 2.05 and 0.0034 at 10^4^ Hz, respectively, and remain stable up to 250 °C. The mechanical properties decrease in turn for PI-FP, PI-FO and PI-FH films due to the increase in free volume fraction. Nevertheless, PI-FH film still exhibits good mechanical properties with a tensile strength of 88.4 Mpa, a tensile modulus of 2.11 GPa and an elongation at break of 4.1%. The correlation between the dielectric and mechanical properties of PI films and their free volume characteristics is well explained with the help of positron annihilation spectroscopy.

## 1. Introduction

With the rapid development of the integration level for ultralarge-scale integration (ULSI) in the semiconductor industry, the size of electronic devices is reducing and the density of transistors is increasing, causing an increase in interconnection delays, cross-talk noise and the power dissipation of circuits [1,2,3,4]. It has become a bottleneck restricting further improvements in the speed of integrated circuits, mainly because the dielectric performance of traditional low dielectric material (SiO_2_) between lines in microelectronic circuits cannot meet the requirements for the dielectric constant (*k*) = 3.9~4.2. Studies show that [5,6,7] some materials with ultra-low *k* are used to replace traditional low-dielectric SiO_2_, which can effectively reduce the interconnection delay, crosstalk and energy consumption. Therefore, in the context of the deep development of ULSI, the development and application of high-performance dielectric materials with low dielectric (*k* ≤ 2.5) and ultra-low dielectric (*k* ≤ 2.0) constants is urgent and inevitable. Such dielectric materials as interlayer dielectrics can reduce the capacitance between the metal interconnections, the resistance capacitance delay, the line-to-line crosstalk noise, and the power dissipation [8,9]. In recent years, polymer materials are considered as a kind of low dielectric materials with good application prospects in ULSI due to their natural low density, good flexibility, low molecular polarizability and natural microporosity. Among these polymer materials, polyimide (PI) films attracted more attention and are widely used as electronic materials due to their low dielectric constants, and good thermal and chemical stability; therefore, they are currently considered as one of the most promising polymer candidates for the next generation of high-performance interlayer dielectrics in microelectronic devices [10,11,12]. However, traditional PIs, such as commercial DuPont Kapton PI films, have a *k* value of 3.2~3.8 [13], which struggles to meet the requirement of ultra-low *k* (≤2.0) for ULSI. Hence, it is significant and urgent to develop new PIs with ultra-low dielectric constants. Many experimental methods are used to reduce the dielectric constant of PIs. Introducing pores can effectively reduce the *k* values and was successfully used to create ultra-low-*k*, accompanied by a loss of the mechanical strength of PIs [5,14,15,16,17]. Therefore, researchers propose designing an appropriate molecular structure to equip low-*k* PIs with low dielectric constants without damaging their excellent intrinsic properties. Fluorine atoms have high electronegativity, which can reduce the conjugation of electron clouds in the molecular structure, in turn reducing the molecular polarizability. Therefore, introducing fluorine atoms into the main chain and increasing fluorine content to prepare PI films with low *k* values is widely reported [3,8,18,19,20,21]. However, studies show that there are some limitations in reducing the *k* value of polyimide only by increasing the fluorine content. When *k* decreases to 2.4, the further decrease in *k* is not clear with the increasing fluorine content [22]. At the same time, with the increase in fluorine content, the hydrogen fluoride generated at a high temperature causes more serious corrosion to inorganic materials [23], and the adhesion and mechanical strength of materials weaken to a certain extent. In addition, substituent group with large volume can hinder the dense stacking of molecular chains, then increase the free volume fraction of polymers, thus reducing the number of polarized molecules per unit volume [24,25]. Therefore, diamines or dianhydride with macromolecular groups are often used to synthesize PIs with low dielectric constant. 

Positron annihilation spectroscopy, as a means of solid defect detection [26], was widely used in the study of defects (including defect vacancies, dislocations, vacancy clusters, holes, etc.) in metals, semiconductors, ceramics and other materials. In porous materials, a fraction of the positrons injected from the positron source formed positronium, which was annihilated from the para state (*p*-Ps, singlet spin state) by emitting two γ-rays with a lifetime of ∼125 *p*s or annihilated from the ortho state (*o*-Ps, triplet spin state) by emitting three γ-rays, with a lifetime 142 ns (in a vacuum environment). The formation probability for *p*-Ps and *o*-Ps is 1:3. The *o*-Ps will pick off one electron from the surrounding molecules and annihilate with a typical value of 1–5 ns in local free volumes, due to collisions with molecules. The pick-off annihilation lifetime of *o*-Ps is highly sensitive to the size of the pores. The *o*-Ps can accurately detect the ultra-micropores because the size of *o*-Ps is only 1.06 Å, much smaller than that of all gas molecules. In addition, positron annihilation spectroscopy is not limited by open or closed pores, which is a powerful means to study porous polymer materials, and played a huge role in the study of free volume pores and pore structure in polymers [27].

In this work, tree fluorinated PI films were prepared by polycondensation reaction using 6FDA as the dianhydride monomer, and PDA, ODA, and HFPBDA as the diamine monomer, respectively. The effects of diamine monomer structure on optical, dielectric and mechanical properties were investigated. The correlation between the dielectric and mechanical properties of PI films and their free volume characteristics was well explained with the help of unique experimental probes such as positron annihilation spectroscopy.

## 2. Materials and Methods

### 2.1. Materials and PI Film Fabrication

**Figure 1:**
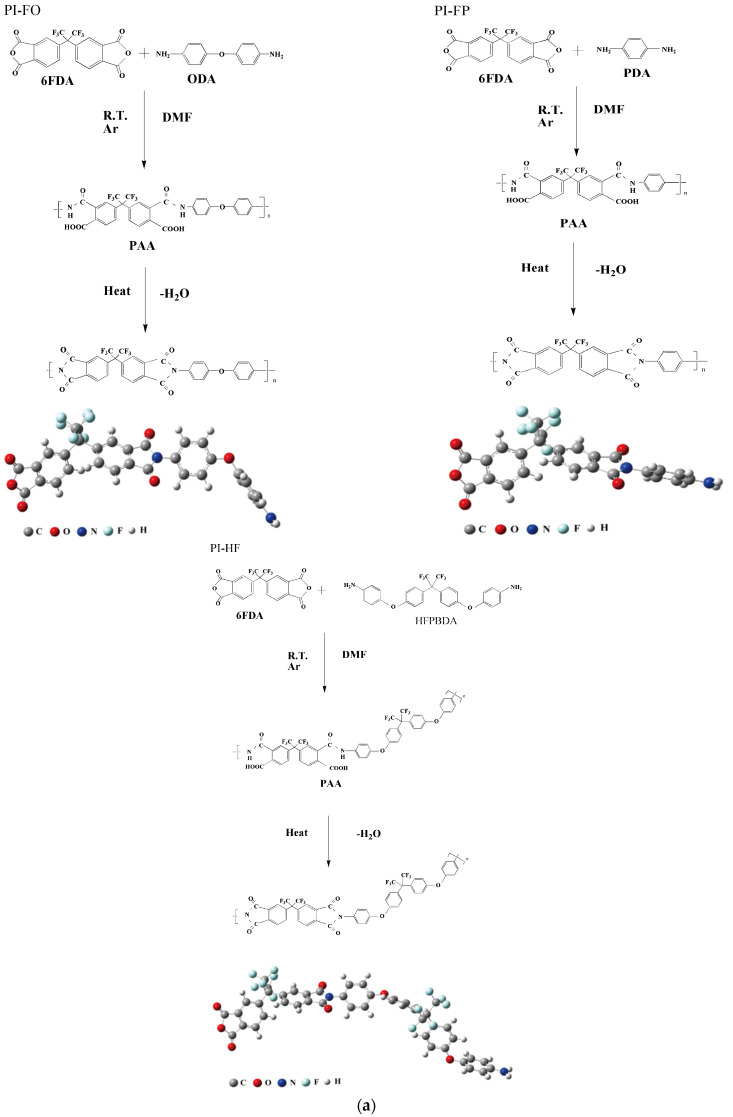

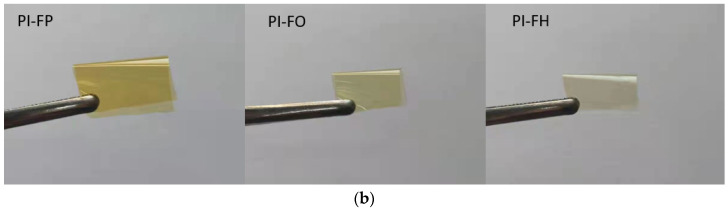
The synthesis procedure for PPA (**a**) and the photos of the PI films (**b**).

6FDA (99%), PDA (99%), ODA (98%), HFBAPP (97%) and *N*,*N*-Dimethyl- form amide (DMF) (≥99.9%) were bought from Aladdin Industrial Corporation (Shanghai, China). A certain proportional amount of diamine (PDA, ODA or HFPBDA) in proportion was weighed according to the solid content of 15% and dissolved in 20 mL DFM in a dried 50 mL flask filled with argon. After the diamine was completely dissolved, 6FDA were weighed in proportion and added into the diamine solution. Then, the flask was put into the ice water mixture, and the magnetic stirrer was used to stir the solution for 8 h, after the polymerization, a viscous, homogeneous, transparent, light-yellow polyamic acid (PAA) solution was obtained. The synthesis reaction process and the photos of the PI films are shown in Figure 1. Finally, after coating on to clean glass plates, the PPA solution was heated at 300 °C for 2 h under vacuum to thoroughly remove the solvent. Three kinds of PI films were thus obtained and the thickness measured ranged from 0.019–0.021 nm. The films were coded PI-FP, PI-FO and PI-FH, where PI-FP represented 6FDA-PDA, PI-FO represented 6FDA-ODA and PI-FH represented 6FDA- HFBAPP, respectively. 

### 2.2. Characterization

The microstructures were characterized by flourier transform infrared spectroscopy (FT-IR) spectra recorded in the transmittance mode on a spectrometer (VERTEX70, Bruker Company, Rudolf-Plank-Str. 27. Ettlingen, Germany). Positron annihilation lifetime spectra were measured using a conventional fast-fast coincidence lifetime spectrometer (Ortec Company, Oak Ridge, TN, USA) with a time resolution of 220 ps and a channel width of 13.0 ps. A ^22^Na positron source with the activity of ~20 μCi sealed between Kapton films was sandwiched between two identical samples (The superposition thickness of PI films on each side of positron source is about 1 mm.) measurements and each spectrum contains more than 1.5 × 10^6^ counts and the obtained lifetime spectra were resolved using PATFIT software package. The transmittance spectra of Ultraviolet-visible for PI films in the wavelength range of 200~800 nm were measured using a UV-VIA-NIR (UH4150, Hitachi Company, 6-6, Marunouchi 1-chome, Chiyoda-ku, Tokyo, Japan) spectrometer. The dielectric properties of the PI films were determined using an Agilent 4294A Precision Impedance Analyzer and Dielectric temperature spectrum measurement system (DMS2000, Partulab company, No. 602, building 9, Guanggu New Power Industrial Park, Gaoxin Road 5, Donghu New Technology Development Zone, Jiangxia District, Wuhan, China). The *k* value was calculated according to the equation: $k=\frac{Cd}{k_{0}S}\;$, where *C* is the capacitance value, $k_{0}$ is the dielectric constant of vacuum which is 8.85 × 10^−12^ F/m; *S* and *d* are the electrode area and thickness of the films. The electric breakdown properties were tested by Voltage-dependent Resistor Characteristic (HG2516, Changzhou Yangzi Electronic Comany, Qingyang Road 2, Xinbei District, Changzhou, China), and the mechanical properties by an electronic universal test machine (UTM 2202, Shenzhen Suns Technology Stock Company, Heli Road 5, Shenzhen, China), and the deformation rate in tensile tests is 10 mm/min.

## 3. Results and Discussion

### 3.1. FT-IR Spectra and EDX

**Figure 2:**
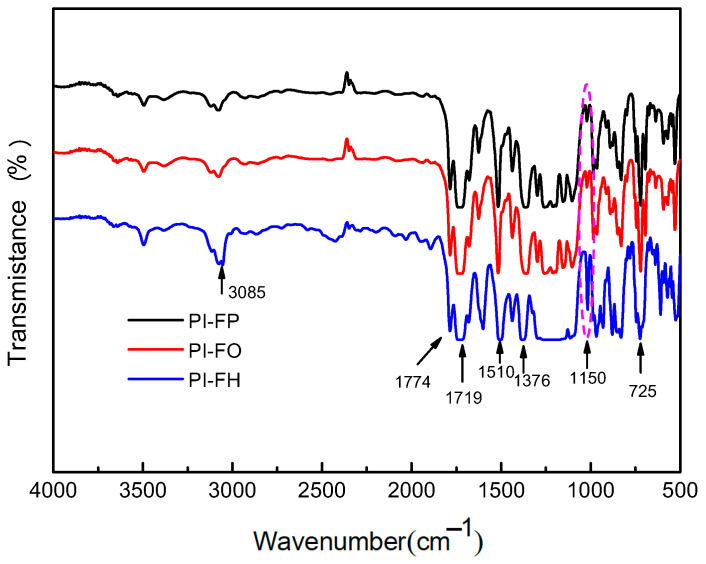
FT—R spectra of PI films.

**Figure 3:**
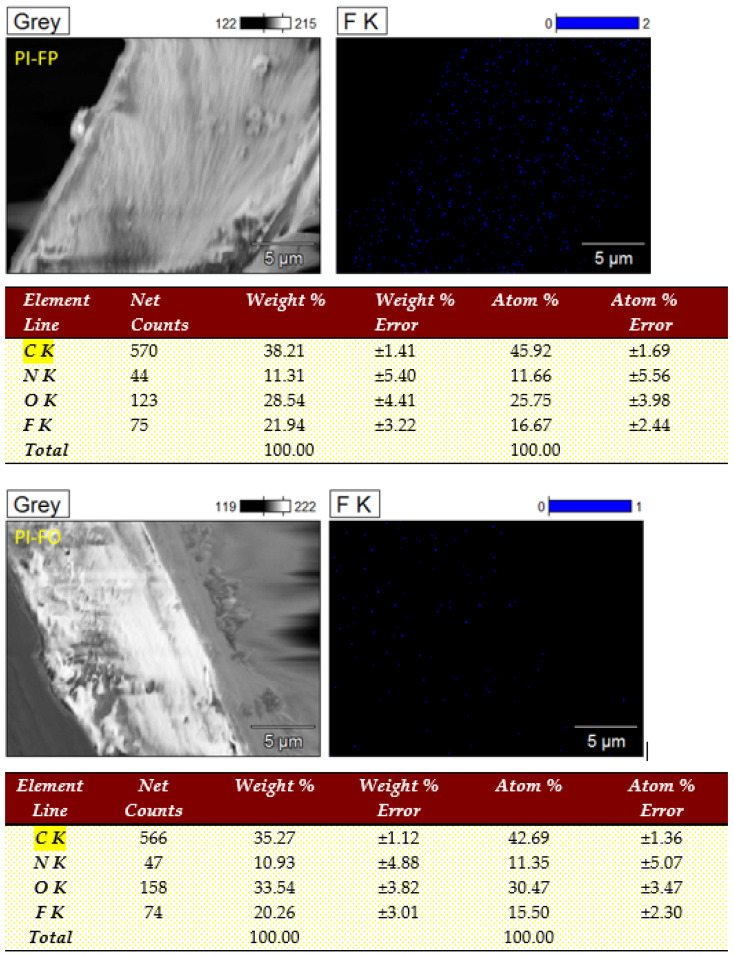

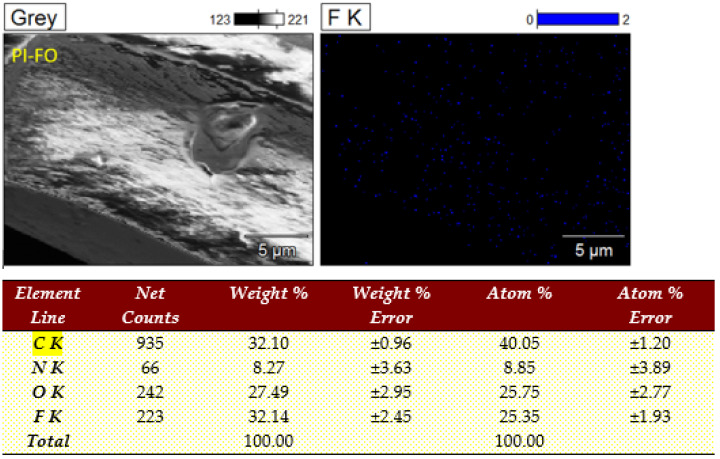
EDX of PI films.

FT-IR spectra of PI films are presented in Figure 2. All of the PI films show characteristic imide absorption peaks, which are consistent with the results reported in the relevant studies [11,12,21]. The peak at 725 cm^−1^ represents the bending vibration of C=O in the imide ring. The peak at 1376 cm^−1^ represents the stretching vibration of C–N in the imide ring. The peaks at 1719 cm^−1^ and 1774 cm^−1^ represent the symmetrical stretching vibrations and the asymmetrical carbonyl stretching vibrations of C=O, respectively, in the imide ring. The peak at 1510 cm^−^^1^ represents the stretching vibration peak of the ben-zene ring, and the peak at 3072 cm^−^^1^ represents the stretching vibration of C-H on the benzene ring. The FT-IR spectra results indicate the successful reaction between the dia-mine monomers (PDA, ODA and HFPBDA) and the dianhydride (6FDA), as well as the complete imidization reaction of PAA. In addition, the C-F stretch vibration absorption peak from the trifluoromethyl (-CF3) groups is observed near 1150 cm^−1^ (within the pink dotted line), and the absorption intensity of PI-FH is much stronger than that of PI-FP and PI-OP due to more -CF3 groups in the molecular chain unit of PI-FH. The EDX results of PI films are shown in Figure 3; it can be seen that the F element content is 21.94 wt%, 20.26 wt% and 32.14 wt% for PI-FP, PI-FO and PI-FH, respectively. 

### 3.2. Positron Annihilation Analysis

The discrete analysis method is widely used to analyze the positron lifetime spectrum. In this analysis, the ideal positron lifetime *L*(*t*) is the superposition of multiple exponential positron components, which can be expressed by the following formula [28]:

(1) $$L\left(t\right)=\sum_{i=1}^{n}I_{i}\exp\left(-t\lambda_{i}\right)\;$$

where *n* is the number of components; $I_{i}$ is the initial intensity of component *I*; *t* is time(channel number); $\lambda_{i}$ is the annihilation rate of component *I*, whose reciprocal (1/$\lambda_{i}$) is the average life $\tau_{i}$ of component *I*. In the actual measurement, considering the time resolution of the instrument, the positron lifetime spectrum (*Y*(*t*)) obtained becomes the convolution of the ideal lifetime spectrum and the instrument resolution function:

(2) $$Y\left(t\right)=L\left(t\right)\cdot R\left(t\right)=N_{t}\sum_{i=1}^{\infty }I_{i}\int_{0}^{\infty }R\left(t-t^{\prime }\right)\exp(-\lambda_{i}t^{\prime })d\;t^{\prime }+B$$

where $N_{t}$ and *B* are the total count and the background count of life spectrum; R(t) is the resolution function of the lifetime spectrometer; $R\left(t\right)=\frac{1}{\sigma \sqrt{\pi }}e^{-{\left(\frac{t}{\sigma }\right)}^{2}}\;$, $\;\sigma =\frac{FWHM}{2\sqrt{2}}\;$, σ is standard deviation; and FWHM is full width at half maximum of the Gaussian function. Based on Equation (2), the positron lifetime spectrum can be fitted by the least square method to obtain $\lambda_{i}$ and $I_{i}$. At present, PATFIT is a widely used spectrum resolution software package, in which POSITRONFIT can be used to obtain the lifetime $\tau_{i}\;$ and its intensity $I_{i}$.

**Figure 4:**
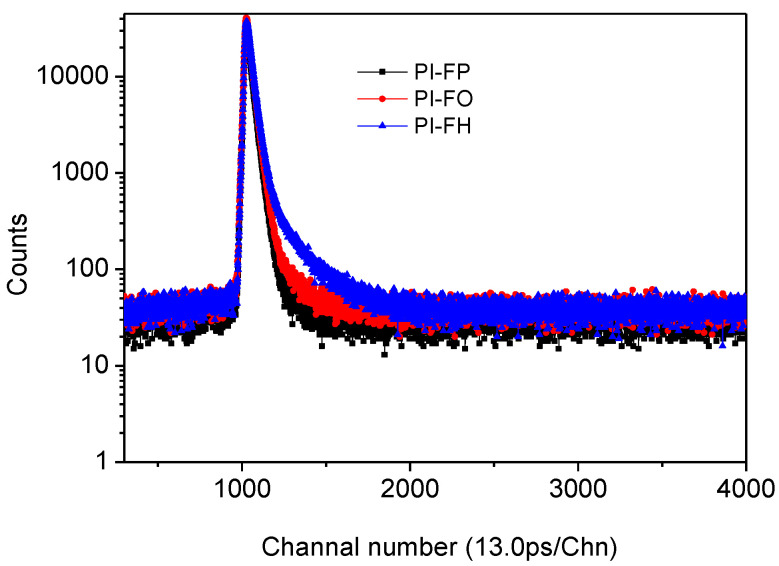
Positron life spectrum of the PI films.

The positron lifetime spectra for PI films are shown in Figure 4 and the detailed positron life time parameters are listed in Table 1, in which there are three lifetime components (*τ*_1_, *τ*_2_ and *τ*_3_) and their corresponding intensities (*I*_1_, *I*_2_ and *I*_3_) for PI-FP, PI-FO and PI-FH films, respectively. The *τ*_1_ is derived from the *p*-Ps lifetime and the free annihilation lifetime of the positrons. The *τ*_2_ (424–444 ps) is due to the annihilation of free positrons trapped in vacancy-type defects on the polymer chains or free volume defects (cavities or vacancies or low-electron-density sections) of PI films without forming the metastable bound state, i.e., Ps. The τ_3_ in the range of 2031 ps to 2246 ps corresponds to the pick-off annihilation of *o*-Ps in the free volume of the amorphous phase for the PI films [27,29]. The probability of the pick-off annihilation of *o*-Ps is related to the size of the free volume. Therefore, the lifetime of *o*-Ps is also directly related to the size of free volume that can be estimated by the Tao-Eldrup model [30]:

(3) $$\tau_{3}=0.5ns{\left[1-\frac{R}{R+∆R}+\frac{1}{2\pi }\sin\left(\frac{2\pi R}{R+∆R}\right)\right]}^{-1}$$

where *τ*_3_ is the annihilation lifetime of *o*-Ps; the prefactor of 0.5 ns is the spin-averaged Ps annihilation lifetime, which is also observed in densely packed molecular crystals [8]; *R* is the radius of voids; Δ*R* is the empirical electron layer thickness parameter around the voids, normally taken as 0.1656 nm. The intensity (*I*_3_) of the *o*-Ps pick-off annihilation is closely related to the free volume fraction of polymer. The free volume fractional $FFV_{PALS}$ can be estimated by the semi-empirical equation [31]:

(4) $$FFV_{PALS}=CV_{f}I_{3}\;$$

where *C* is a material-dependent constant which is defined as 0.0018 Å^−3^ [31,32,33]; $V_{f}=\frac{4}{3}\pi R^{3}$; $I_{3}$ is the intensities of $\tau_{3}$. The free volume parameters of the PI films calculated according to Equations (3) and (4) are given in Table 2. The free volume fraction of PI-OP is almost the same as that of PI-FP. However, that of the PI-FH film is significantly higher than that of the former two films.

The characteristics of the free volume defect are closely related to the molecular chain structure. The calculated results of free volume parameters for the three PI films indicate that the chain length of molecular units and the number of -CF_3_ groups in the molecular chain unit have a significant effect on the free volume size and the free volume fraction of polyimide. A long molecular chain unit with more benzene rings can inhibit the dense stacking of molecular chain because the adjacent benzene rings form a non-coplanar structure due to the repulsion between the π bonds. In addition, the -CF_3_ group with large molar volumes can also effectively inhibit the dense stacking of molecular chain. Therefore, PI-FH has a longer molecular chain unit and more -CF_3_ groups than the other two PIs, which makes PI-FH film have a larger free volume size and higher free volume fraction than other two PI films.

### 3.3. UV-Visible Transmission Spectra

**Figure 5:**
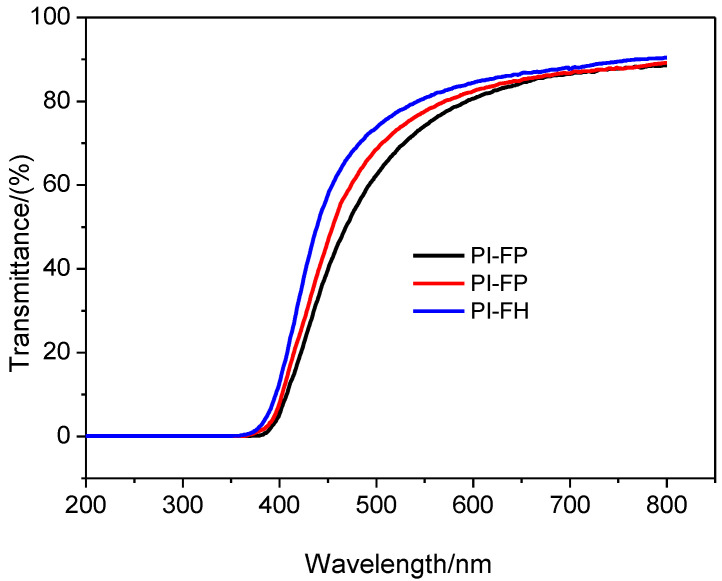
UV—VIS transmittance spectra of the PI films.

The UV-VIS transmittance spectra in the range of 400–650 mm^−1^ of PI-FP, PI-FO and PI-FH are shown in Figure 5, and their highest transmittance is shown in Table 3. The transmittance increase in turn for the three PI films and accordingly, the color gradually fades from orange to light yellow (Figure 1b). In general, the UV-Vis transmittance and coloration of aromatic polymers depends on the formation of their charge transfer complexation (CTC) and/or conjugated aromatic structures [34]. The -CF_3_ has a negative effect on the conjugation degree of aromatic structure. The fluorine atoms with strong ability of electron-withdrawing can inhibit the formation of the intermolecular and intramolecular CTC and increase the transmittance of PI [35].

**Figure 6:**
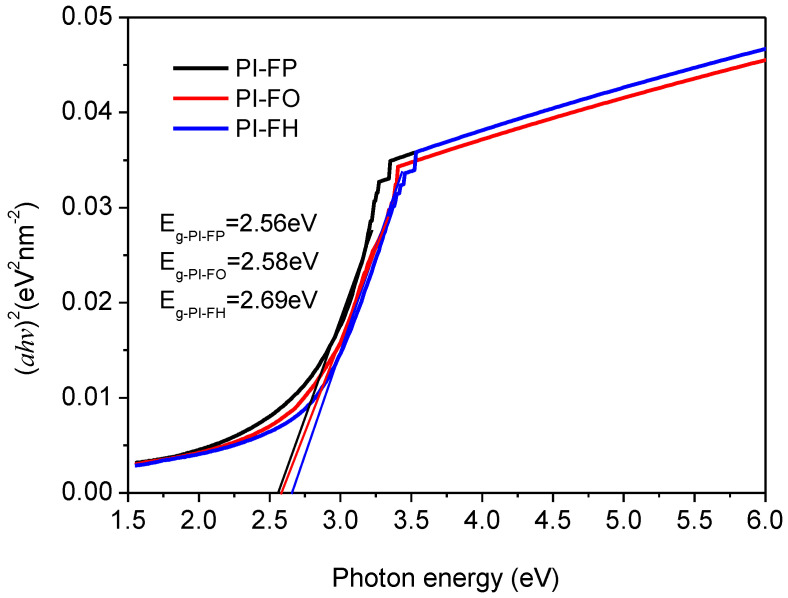
The relation between absorption coeffi- cient spectra and the energy gap.

In addition, the energy gap (*E*_g_) is an important optical parameter, which can be obtained (according to Figure 6) by Equation (5):

(5) $${\left(\frac{\alpha h\nu }{K}\right)}^{1/2}=h\nu -E_{g}$$

where *α* is the absorption coefficient, which can be obtained in terms of: *α* = (1/*d*) ln(1/*T*), *d* is the film thickness, *T* is the transmission, *K* is a coefficient, *h* is the Planck constant, and *ν* is the photon frequency. The *E*_g_ value can be obtained based on the linear approximation of the absorption edge to the energy axis, i.e., the intercept at the energy axis. The estimated *E*_g_ value is 2.56 eV, 2.58 eV and 2.69 eV for PI-FP, PI-FO and PI-FH films, respectively.

The effect of the conjugation degree of the aromatic structure on the optical property of the polymer can be determined by the energy gap, since the length of conjugation within the polymer chain is related to the *E*_g_. The -CF_3_ group with a large volume located on the side of the polymer chain leads to the torsion of molecular chains and reduces conjugation length in the molecular chain, thus reducing the conjugation absorption. In addition, the oxyether (-O-) bond can break the conjugation of the molecular chain and weaken the large π conjugation absorption of the aromatic polymer chain. Therefore, the increase in the number of -CF_3_ groups and oxygen ether bonds in the molecular chain can lead to the increase in *E*_g_.

### 3.4. Dielectric Properties

**Figure 7:**
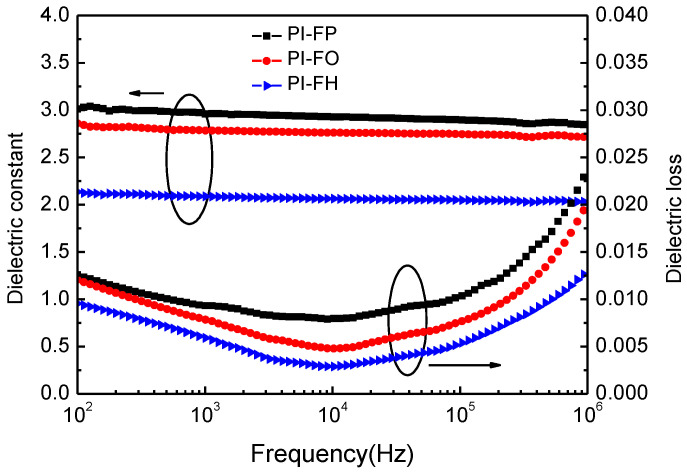
The dependence of the dielectric properties on frequency for PI films at room temperature.

The dependence of the dielectric properties on the frequencies of PI films at room temperature is shown in Figure 7, and the measured dielectric constant dielectric loss at 10^4^ Hz is listed in Table 4. The films exhibit low dielectric constants (*k*) of 2.06–2.92 at 10^4^ Hz, which are much lower than the dielectric constant of a commercial PI film (3.49 at 10^4^ Hz). The dielectric constant of PI-FO is only slightly lower than that of PI-FP. However, the dielectric constant of PI-FH film is much lower than that of the former two films.

The dielectric constant mainly depends on the number of polarized molecules per unit volume of polymer [26,36]. The relation between the dielectric constant and polarizability can be explained by the Debye equation [21].

(6) $$\frac{k-1}{k+2}=\frac{4}{3}\pi N\left(a_{e}+a_{d}+\frac{\mu^{2}}{3k_{b}T}\right)\;$$

where k is dielectric constant, N is the number density of dipoles, $a_{e}$ the electric polarization, $a_{d}$ is the distortion polarization, μ is the orientation polarization related to the dipole moment, and T and $k_{b}$ are temperature and the Boltzmann constant, respectively.

The influence of -CF_3_ groups located at the side molecular chain on the dielectric properties of PI includes two aspects. On the one hand, -CF_3_ groups can hinder the dense stacking behavior of molecular chains, increase the free volume fraction, and then reduce the number of polarized molecules (*N*) per unit volume. On the other hand, less bond- conjugated electrons can lead to the reduction in $a_{e}$,$\;a_{d}$ and μ due to the strong ability of the electron-withdrawing of fluorine atoms [21]. The free volume fraction of PI-FO is slightly higher than that of PI-FP, resulting in a higher dielectric constant of PI-FO than that of PI-FP. Compared with PI-FP and PI-FO films, the PI-FP film presents the lowest dielectric constant (*k =* 2.05) due to the most -CF_3_ groups contained in the molecular chain units of PI-FP. According to the definition of ultra-low dielectric constant (≤2.0) material, the PI-FP film can be approximately regarded as an ultra-low dielectric PI film.

**Figure 8:**
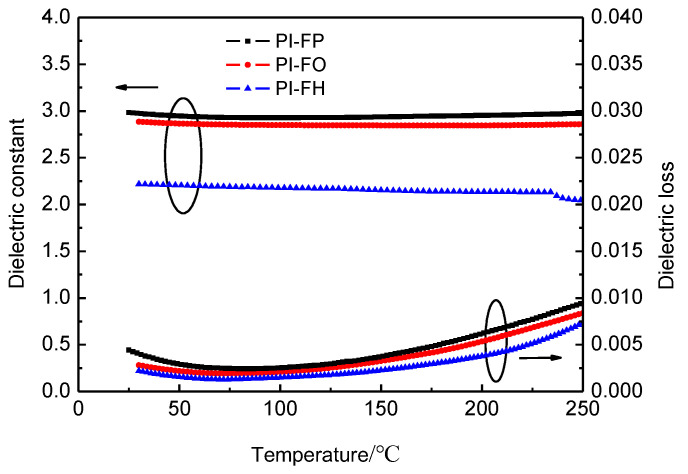
The temperature dependence of the dielectric properties at 10^4^ Hz for PI films.

In addition, low dielectric loss (*D*) is an important index to evaluate the dielectric properties of dielectric materials. Generally, the total energy loss in dielectric materials including the loss from the dielectric relaxation process and loss from DC conductivity can be expressed as Equation (7) [37]:

(7) $$D=\frac{\omega^{2}\varepsilon_{0}\tau \left(\varepsilon_{s}^{\prime }-\varepsilon_{\propto }^{\prime }\right)+\left(1+\omega^{2}\tau^{2}\right)\sigma_{dc}}{\omega \varepsilon_{0}\left(\varepsilon_{s}^{\prime }+\varepsilon_{\propto }^{\prime }\omega^{2}\tau^{2}\right)}\;$$

where *w is* the frequency; $\varepsilon_{s}^{\prime }$ and $\varepsilon_{\propto }^{\prime }$ are the static and high-frequency permittivity; *τ* is relaxation time of the polarized molecule; $\varepsilon_{0}$ is permittivity of the vacuum; and $\sigma_{dc}$ is DC conductivity. At low frequencies, ωτ≪1, the dielectric loss mainly dominated by $\sigma_{dc}$ and at high frequencies, $\sigma_{dc}=0$, the dielectric loss mainly dominated by *τ*. The high free volume fraction is conductive reducing $\sigma_{dc}$, and then reducing the dielectric loss from leakage conduction at low frequencies. The low molecular polarizability is conductive to reduce the dielectric loss at high frequencies from the relaxation of polarization molecular. Therefore, the high free volume fraction and low molecular polarizability are beneficial to reduce the dielectric loss of polymers [38]. PI-FP has extremely low dielectric loss values (*D* = 0.0034) at 10^4^ Hz, which are very important for reducing the energy consumption of devices when applied as an interlayer medium applied in ULSI. The temperature dependence of the dielectric properties at 10^4^ Hz for the three PI films is shown in Figure 8. All the curves of the dielectric constant and loss versus temperature display a very good stability of up to 250 °C. The excellent temperature stability of dielectric properties suggests a good potential for microelectronic applications as dielectric and insulating materials.

The electrical breakdown strength is an important parameter to evaluate the insulating performance of dielectric materials. Because breakdown strength can lead to a short circuit which seriously damages the electronic devices. The measured DC breakdown strength values of the three PI films are shown in Table 4. The breakdown strength decreases successively, which can be ascribed to the gradual increase in the average size of free volume and the fraction of free volume for PI-FP, PI-FO and PI-FH films.

### 3.5. Mechanical Properties

**Figure 9:**
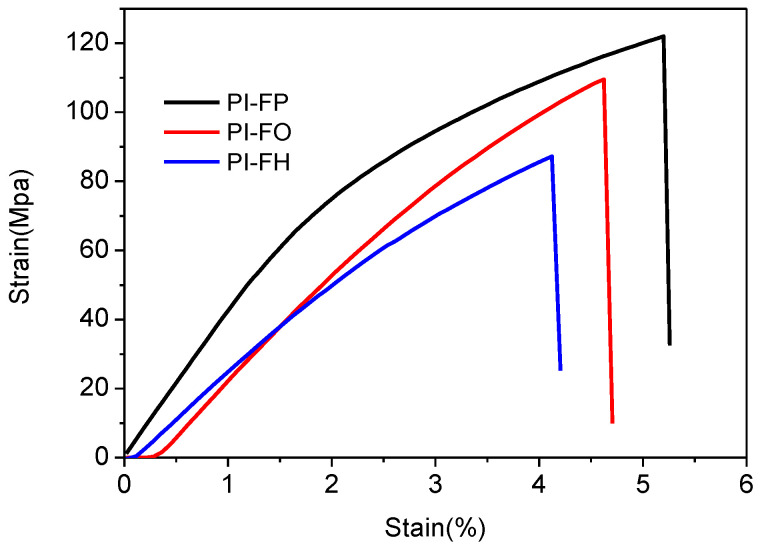
Measured stress versus strain curves of PI films subjected to tensile tests.

The mechanical property of the PI-based film is identified as an important index to evaluate its application as an interlayer material. The measured stress-strain curves of films subjected to tensile tests at room temperature are displayed in Figure 9. The measured tensile strength, tensile modulus, and elongation at break of films are listed in Table 5. The tensile strength decreases gradually from 122.5 MPa for the PI-FP film, to 109.6 MPa for PI-FO film, and to 88.4 MPa for PI-FH film. Accordingly, the tensile modulus decreases gradually from 2.86 GPa for the PI-FP film, to 2.42 GPa for PI-FO film, and to 2.11 GPa for PI-FH film; the elongation at break decreases from 5.1% for the PI-FP film, to 4.6% for the PI-FO film and to 4.1% for the PI-FH film. Generally, the mechanical properties of polymers are affected by many factors, such as free volume defect, structure of molecular chain, crystallinity, test temperature and so on. Firstly, the free volume factor is very important. It is considered that the tensile strength of the PI film decreases with the increase in free volume size and free volume fraction [29]. Secondly, the molecular chain structure factor cannot be ignored. When the -CF_3_ groups are introduced into the molecule chain, the electron cloud of the C-C bond will transfer to the side of the C atom close to -CF_3_ due to the strong electron-withdrawing ability of fluorine atoms, which reduces the bond energy of C-C bond, and then reduces the tensile strength. In addition, the bond energy of C-O (326 kJ/mol) is slightly lower than that of C-C (332 kJ/mol), which is the reason in the decline of mechanical properties of PI-FO compared with PI-FP. Therefore, according to the free volume parameters and the structure of molecular chain units of the three PI films, it is not difficult to understand that the mechanical properties decrease in turn for PI-FP, PI-FO, and PI-FH films. In addition, the existence of an -O- bond in the molecular chain increases the flexibility of the molecular chain and reduces the tensile modulus of the PI film [25]. Therefore, the tensile modulus decreases in turn for PI-FP, PI-FO, and PI-FH films. Nevertheless, it is worth noting that the PI-FH film still exhibits good mechanical properties compared with the other fluorinated PI films, as reported in studies [20,39] on the case of excellent dielectric properties (Figure 9).

## 4. Conclusions

In summary, three PI films were synthesized by means of polycondensation, taking 6FDA, PDA, ODA and HFPBDA as raw materials. The optical and dielectric properties of PI film were improved by changing of diamine monomer structure. Among the three PI films, the PI-FH film presents the best optic transmission (highest transmittance = 90.2%) and the best dielectric properties (*k* = 2.05, *D* = 0.0034 at 10^4^ Hz). Compared with PDA and ODA residues, HFPBDA residue has more -CF_3_ groups and a longer chain length. The -CF_3_ groups can effectively weaken the intermolecular and intramolecular CTC effect and the conjugation degree of the aromatic structure, and then increase the optic transmission and energy gap. Compared with PDA and ODA residues, the HFPBDA residue in the molecular chain can more effectively increases the free volume fraction of PI, and then reduce the number of polarized molecules per unit volume. Additionally, the -CF_3_ can reduce the polarizability due to the strong electron-withdrawing ability of fluorine atoms. This is the main reason that the dielectric properties of PI-FH film are the most improved. The mechanical properties weaken in turn for PI-FP, PI-PO and PI-FH films. Nevertheless, the PI-FH film still exhibits a tensile strength of 88.4 Mpa at room temperature considering its excellent dielectric properties. This study establishes a novel method of understanding the dielectric and mechanical properties of PI films based on their free volume defect characteristics and the correlation between them, aided by positron annihilation spectroscopy.

## Figures and Tables

**Table 1:** The positron life time parameters of PI films.

PI Films	*τ*_1_/ps	*τ*_2_/ps	*τ*_3_/ps	*I*_1_/%	*I*_2_/%	*I*_3_/%
PI-FP	210.5 ± 4.6	429.6 ± 3.7	2031.4 ± 30.3	36.4 ± 2.3	58.6 ± 2.1	5.0 ± 0.6
PI-FO	220.6 ± 5.4	424.6 ± 3.5	2059.3 ± 28.2	32.7 ± 1.4	61.0 ± 1.3	5.9 ± 0.8
PI-FH	225.4 ± 9.0	444.9 ± 9.2	2246.2 ± 29.9	30.6 ± 3.6	59.7 ± 3.5	9.7 ± 1.4

**Table 2:** The free volume hole parameters of PI films.

PI Films	*R/*Å	*V_f_/*Å^3^	*f_r_*/%
PI-FP	2.88 ± 0.02	99.76 ± 2.83	0.90 ± 0.02
PI-FO	2.90 ± 0.03	102.39 ± 2.68	1.09 ± 0.02
PI-FH	3.06 ± 0.02	120.48 ± 2.96	2.10 ± 0.04

**Table 3:** The cut-off wavelength and highest transmittance of PI films.

Films	Highest Transmittance/%	*E*_g_/eV
PI-FP	88.5 ± 2.0	2.56
PI-FO	88.8 ± 2.3	2.58
PIF-H	90.2 ± 2.1	2.69

**Table 4:** The dielectric parameters of PI films.

Films	*k*/10 kHz	*D*/10 kHz (10^−^^2^)	Electrical Breakdown (kV/mm)
PI-FP	2.92 ± 0.08	0.83 ± 0.03	65.4 ± 2.8
PI-FO	2.76 ± 0.07	0.52 ± 0.03	61.3 ± 2.5
PI-FH	2.05 ± 0.07	0.34 ± 0.03	51.6 ± 2.1

**Table 5:** The tensile strength, tensile modulus, and elongation at break of PI films.

Films	Tensile Strength/MPa	Tensile Modulus/GPa	Elongation at Break (%)
PI-FP	122.5 ± 3.7	2.86 ± 0.08	5.1 ± 1.4
PI-FO	109.6 ± 3.4	2.42 ± 0.07	4.6 ± 1.2
PI-FH	88.4 ± 3.1	2.11 ± 0.05	4.1 ± 0.9

## Data Availability

Not applicable.
